# A Non-Volatile Tunable Ultra-Compact Silicon Photonic Logic Gate

**DOI:** 10.3390/nano12071121

**Published:** 2022-03-28

**Authors:** Zheng Peng, Junbo Feng, Huan Yuan, Wei Cheng, Yan Wang, Xiaodong Ren, Hao Cheng, Shengyin Zang, Yubei Shuai, Hao Liu, Jiagui Wu, Junbo Yang

**Affiliations:** 1 College of Artificial Intelligence, Southwest University, Chongqing 400715, China; pengzheng97@email.swu.edu.cn (Z.P.); huanyuan1806@email.swu.edu.cn (H.Y.); chengmeet@email.swu.edu.cn (W.C.); wangyan1999@email.swu.edu.cn (Y.W.); renxiaod@email.swu.edu.cn (X.R.); chenghao997@email.swu.edu.cn (H.C.); a5163602@email.swu.edu.cn (S.Z.); shuaiyubei@email.swu.edu.cn (Y.S.); liuhao413@email.swu.edu.cn (H.L.); 2 Center of Material Science, National University of Defense Technology, Changsha 410073, China; 3 United Microelectronics Center Co., Ltd., Chongqing 401332, China; junbo.feng@cumec.cn; 4 School of Physical Science and Technology, Southwest University, Chongqing 400715, China

**Keywords:** logic gate, phase change material, inverse design

## Abstract

Logic gates, as one of the most important basic units in electronic integrated circuits (EICs), are also equally important in photonic integrated circuits (PICs). In this study, we proposed a non-volatile, ultra-compact all-photonics logic gate. The footprint is only 2 μm × 2 μm. We regulate the phase change of optical phase change materials(O-PCMs) Sb_2_Se_3_ to switch the function of the logic gate. The Sb_2_Se_3_ possess a unique non-volatile optical phase change function; therefore, when Sb_2_Se_3_ is in the crystalline or amorphous state, our device can work as XOR gate or AND gate, and our designed logic ‘1’ and logic ‘0’ contrasts reach 11.8 dB and 5.7 dB at 1550 nm, respectively. Compared with other traditional optical logic gates, our device simultaneously has non-volatile characteristics, tunability, and additionally an ultra-small size. These results could fully meet the needs of fusion between PICs and EICs, and developing truly chip-scale optoelectronic logic solution.

## 1. Introduction

In recent years, large-scale photonic integrated systems have been confirmed to have a higher speed, higher capacity, and lower power consumption when performing computing tasks, which is suitable for the development of next-generation computing platforms [1,2]. In EICs, a field programmable gate array (FPGA) consists of a large number of logic units. Due to its flexible programmability and non-volatility, it has been widely used in electronics and communications. In PICs, a non-volatile tunable logic gate is also essential for implementing similar integrated devices. On the other hand, the all-optical logic gate is a key device for realizing optical communication networks, optical computing, and optical signal processing, it has been extensively studied in recent years. Many schemes have been proposed to realize all-optical logic gates, such as fibers [3,4,5], semiconductor optical amplifiers [6,7,8], photonic crystals [9,10,11,12,13], and Mach–Zehnder interferometers [14]. However, the functions of the logic gates implemented by these schemes are fixed and cannot be regulated.

The size of an optical logic gate based on the above schemes is generally tens to hundreds of square microns, being a significant fusion barrier between PICs and EICs. For example, in Ref. [15], the photonic crystal method was used to realize an all-optical logic gate with a size of 252 μm^2^. In Ref. [16], also using the photonic crystal method, a gate with a size of 729 μm^2^ was realized. In contrast, we used the inverse design method to realize a device with an ultra-small size; this method has been widely used in photonic devices design [17,18], such as in power splitters [19,20], focusing wavelength demultiplexers [21,22], and gratings [23,24]. Compared with traditional design methods, an inverse design method can search a larger parameter space to obtain high-performance and more compact photonic devices. To meet the needs of the development of photonic integrated circuits, many heuristic algorithms have been applied to an inverse design, including direct binary search algorithm (DBS) [25,26,27,28], genetic algorithm [29,30], particle swarm algorithm [31,32], objective-first algorithm [33,34], and neural networks [35,36,37].

O-PCMs are considered to be promising candidate material for designing reconfigurable photonic devices and memory units [38,39,40]. This is because their optical properties, such as the refractive index change drastically in a non-volatile manner. Devices based on O-PCMs have received signification research attention. They have been investigated for optical switches [41,42,43,44], metalens [45,46,47], mode converters [48,49], and optical neural networks [50,51]. In addition, O-PCMs have been used for memory cells due to their characteristics of fast phase transitions [52]. Experiments have shown [53] that the non-volatile phase transitions of O-PCMs between the crystalline and amorphous states can be controlled using light or electricity. Furthermore, Ge_2_Sb_2_Se_5_ (GST) [54] is one of the widely used non-volatile O-PCMs. The complex refractive index of GST in the amorphous and crystalline states is 4.6-i0.12 and 7.45-i1.49 at 1550 nm, respectively. However, the extinction coefficient of GST crystalline state is too large, which could result in excessive loss. Sb_2_Se_3_ [55,56,57], as a new type of O-PCMs, it’s refractive index will change a lot during the transition between crystalline and amorphous states. More importantly, it has ultra-low loss in the commercial C-band. For example, at 1550 nm, the complex refractive index of Sb_2_Se_3_ in the amorphous and crystalline states is 3.285-i0 and 4.050-i0, respectively, where the imaginary parts are zero for both states, indicating ignorable light absorption. Both crystalline and amorphous states show ultra-low loss, being a rare feature among many O-PCMs.

In this study, we designed and demonstrated a non-volatile, tunable, ultra-compact optical logic gate on a silicon-on-insulator (SOI) platform based on the DBS algorithm and Sb_2_Se_3_. The footprint of the device is only 2 μm × 2 μm. For our device, when Sb_2_Se_3_ is in the crystalline state, the XOR gate is realized, and when Sb_2_Se_3_ is in the amorphous state, it can be used as an AND gate. The function of the device can be regulated by switching the state of Sb_2_Se_3_. As far as we know, most existing optical logic gates have a large footprint, which is not conducive for integration, and their function cannot be controlled. Here, we first propose a tunable ultra-compact silicon optical logic gate, which can be used as a logic gate in future photonic integrated circuits.

## 2. Chip Design and Algorithm

**Figure 1:**
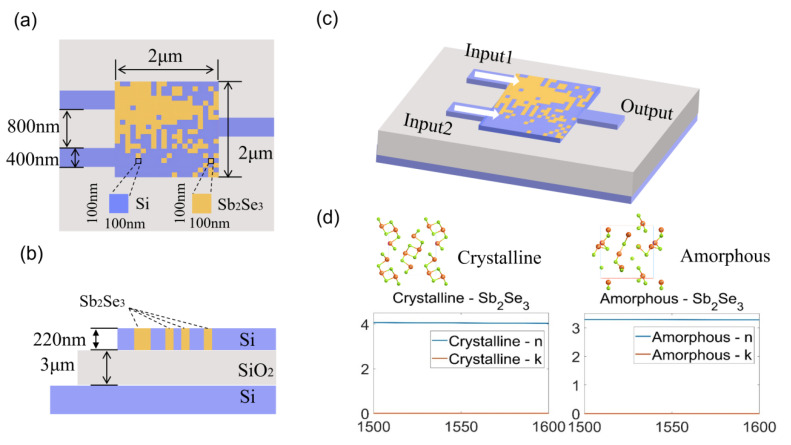
Schematic of the logic gate device. (**a**) Top view of the device; (**b**) Side view of the device; (**c**) Three-dimensional diagram of the device showing two input ports in the left and one output port in the right; (**d**) Atomic distribution and complex refractive index of Sb_2_Se_3_ in crystalline and amorphous states.

The 3D structure, the size of the proposed logic gate and the complex refractive index and atomic distribution of Sb_2_Se_3_ are shown in Figure 1. Our device was based on the SOI platform, where the buried layer was 3 μm silicon dioxide and the top layer was standard 220 nm silicon. Furthermore, Sb_2_Se_3_ was embedded in the top layer silicon. Here, we used silicon instead of Si_3_N_4_ or other materials, mainly considering the complete compatibility with CMOS processes. Moreover, silicon has about a 3.5 refractive index at around 1550 nm and then shows a very strong light field confinement. Before using the DBS algorithm, the 2 μm × 2 μm design area was divided into 20 × 20 square pixels for digital binarization, the size of each pixel was 100 nm × 100 nm. Each pixel has two states, ‘0’ and ‘1’, where ‘0’ represents silicon, and ‘1’ represents Sb_2_Se_3_. The silicon at the ‘1’ positions was etched to a depth of 220 nm and replaced by Sb_2_Se_3_. Compared with the O-PCMs on the surface of silicon, embedded O-PCMs in silicon could have a stronger ability to control light field. With design experience, we set the width of the two input waveguides as 400 nm, the distance between them was 800 nm, and the width of the output waveguide as 400 nm. In Figure 1d, the atomic distribution in the crystalline and amorphous states of Sb_2_Se_3_ and its complex refractive index at 1500–1600 nm are presented. Especially, for Sb_2_Se_3_, its extinction coefficient (k) is 0 in both crystalline and amorphous state. The design goal of our device is that the device should act as an XOR gate when Sb_2_Se_3_ is in the crystalline state and an AND gate when Sb_2_Se_3_ is in the amorphous state.

**Figure 2:**
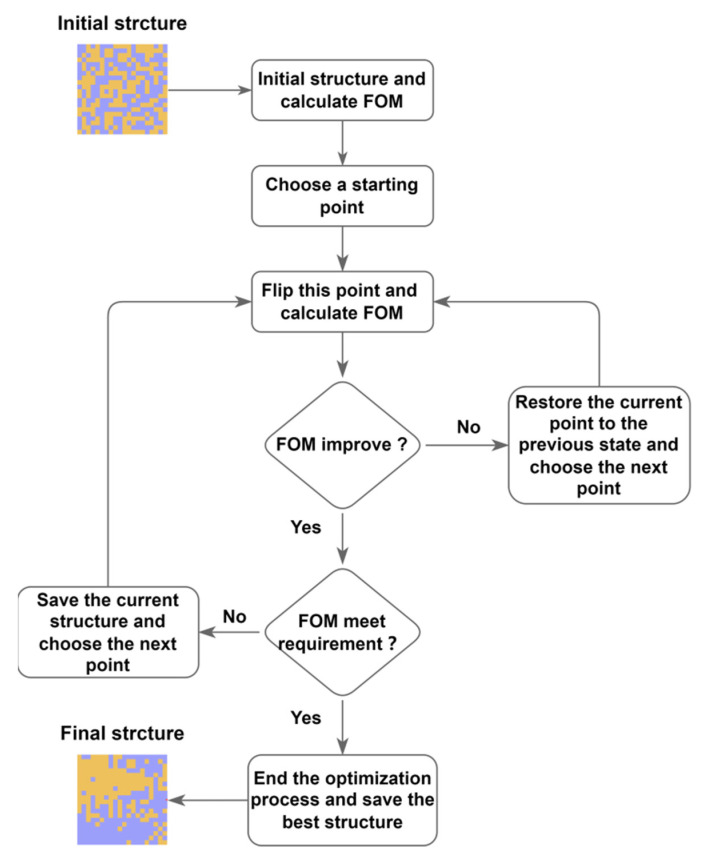
Flow chart of the DBS algorithm. First, a random structure is initialized, and the points in the structure are flipped point by point and the *FOM* was calculated. The structure with a higher *FOM* will be saved, iterating until the stop requirements were met.

The flowchart of the inverse design based on the DBS algorithm is shown in Figure 2. The structure of the design area was represented by a 20 × 20 matrix of 0 s and 1 s. The corresponding spectrum of each structure was obtained after a simulation. We defined a figure of merit (*FOM*) to evaluate the performance of the current structure. In this way, we abstracted the physical optimization objective into a mathematical optimization process. With the DBS algorithm, we optimized the structure and improved the *FOM* to obtain the required device performance. *FOM* was defined as follows:

(1) $$FOM_{C}=\left(P_{C10}+P_{C01}\right)-3\times P_{C11}-\left|P_{C10}-P_{C01}\right|$$

(2) $$FOM_{A}=P_{A11}-1.5\times \left(P_{A10}+P_{A01}\right)-3\times \left|P_{A10}-P_{A01}\right|$$

(3) $$FOM=1.5\times FOM_{C}+FOM_{A}$$

The *FOM* consisted of two parts, *FOM_A_* and *FOM_C_*, as a crystalline and amorphous *FOM*, respectively. Each source was assumed to have four states, i.e., ‘00’, ‘01’, ‘10’ and ‘11’; the first represented the state of input1 and the second represented the state of input2. Furthermore, *P_C_*_10_, *P_C_*_01_, and *P_C_*_11_ in formula (1) are the output intensities when the input states are ‘10’, ‘01’ and ‘11’, respectively, in the crystalline state, and *P_A_*_10_, *P_A_*_01_, and *P_A_*_11_ in formula (2) are amorphous state. During the optimization process, our *FOM* is adjusted to obtain the final form. A 20 × 20 matrix containing 0 s and 1 s was first randomly generated. Then, the 3D FDTD was used to solve Maxwell’s equations and the *FOM* of the device structure was calculated. A starting point in the matrix was selected and its state was flipped (‘0’ to ‘1’ or ‘1’ to ‘0’), and the *FOM* was calculated after the flip. If the *FOM* improved, the flipped state was retained, and if the *FOM* did not improve, the flipped state was restored. This procedure was repeated for each point in the structure (by row or column). After all the points in the matrix are calculated, one iteration ends and a new iteration starts again. The algorithm will continue to run until the target conditions are met.

## 3. Chip Simulations and Analysis

All simulations in this work were conducted with the 3D FDTD analysis software (Ansys Lumerical FDTD 2020 R2.4). In the case that our proposed device functions as an XOR gate, when the source is ‘00’ or ‘11’, the output state is ‘0’, and when the source is ‘10’ or ‘01’, the output state is ‘1’; In cases where the device functions as an AND gate, when the source is ‘10’, ‘01’, or ‘11’, the output state is ‘0’, and when the source is ‘11’, the output state is ‘1’.

We used the contrast ratio (*CR*) to evaluate the degree of difference between the ‘0’ and ‘1’ states of the of logic gate device, where *CR* is defined as:

(4) $$CR=10\times \log\left(P_{1}/P_{0}\right)$$

where *P*_1_ is the minimum intensity with a logical value of ‘1’, and *P*_0_ is the maximum intensity with a logical value of ‘0’.

**Figure 3:**
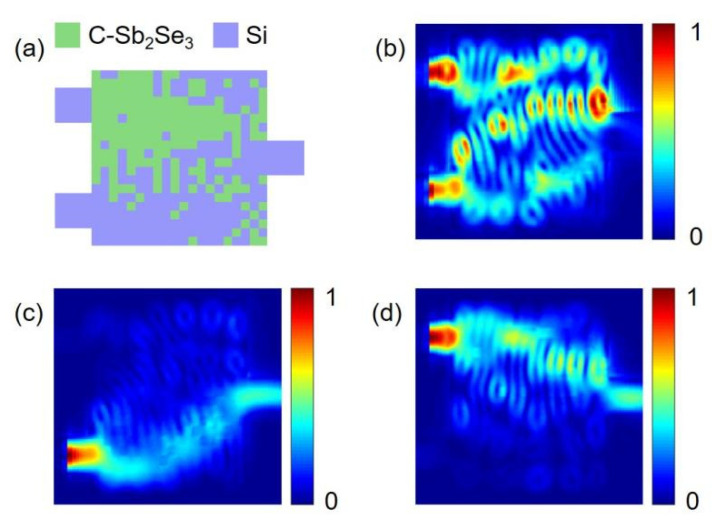
Energy density distribution of the XOR gate when Sb_2_Se_3_ is in the crystalline state. (**a**) The final structure of our device; the green pixels are crystalline Sb_2_Se_3_ and the purple pixels are silicon; (**b**) Energy distribution when the input state is ‘11’; (**c**) Input state is ‘01’; (**d**) Input state is ‘10’.

**Figure 4:**
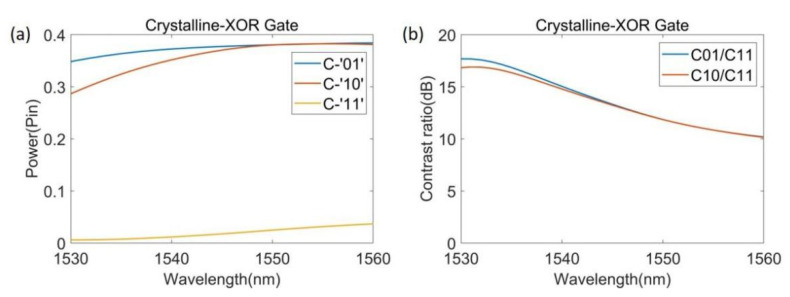
Performance graph of XOR gate. (**a**) Spectral power curve of the XOR gate when Sb_2_Se_3_ was in the crystalline state; (**b**) Contrast ratio curve of the XOR gate.

When the source state is ‘00’, the output state of our device is always ‘0’; hence, only the three states of ‘10’, ‘01’ and ‘11’ are discussed in the following section. Figure 3 presents the optimized structure of our device and the energy density distribution of the three states of ‘10’, ‘01’, and ‘11’ when our device functions as the XOR gate. As shown in Figure 3c,d, when the input state is ‘01’ or ‘10’, the power of the output port is higher, which is the ‘1’ state; when the input state is ‘11’, almost no output power is observed, which is the ‘0’ state. From Figure 3b, it can be seen that after the light of inputs 1 and 2 enters the device, it converges above the output port and cannot escape through the output port, making the output power low. Figure 4 presents the spectral power curve of the XOR gate when Sb_2_Se_3_ is in the crystalline state at 1530 to 1560 nm.

Table 1 shows the output intensity of the XOR gate in each source state when λ = 1550 nm. When the source state is ‘00’, the output intensity is ‘0’; for the source states of ‘10’, ‘01’, and ‘11’, the output intensities and states are 0.381 P_in_ and ‘1’, 0.381 P_in_ and ‘1’, and 0.025 P_in_ and ‘0’, respectively. The *CR* of the XOR gate is 11.8 dB, and we set the threshold between the logics ‘0’ and logic ‘1’ to 0.2 P_in_. In this way, our device can easily distinguish logic ‘0’ and logic ‘1’ and realize the function of an XOR gate.

**Figure 5:**
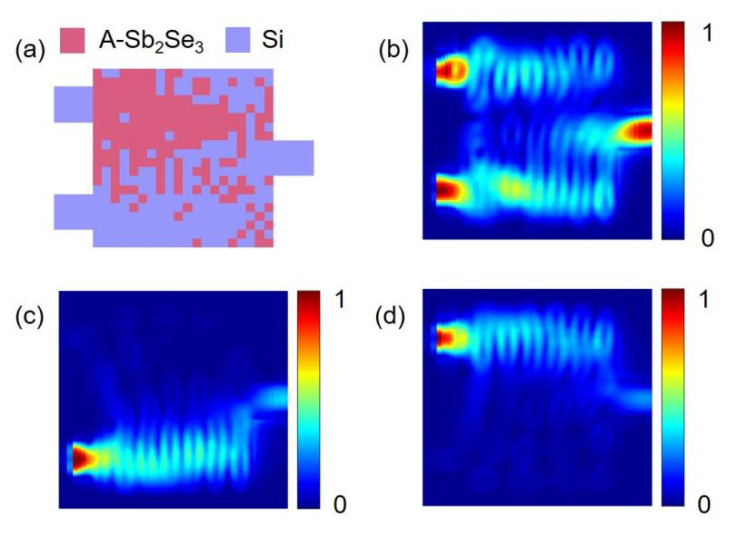
Energy density distribution of the AND gate when Sb_2_Se_3_ is in the amorphous state. (**a**) The final structure of our device; the red pixels are Sb_2_Se_3_ in the amorphous state and the purple pixels are silicon; (**b**) Energy distribution when the input state is ‘11’; (**c**) Input state is ‘01’; (**d**) Input state is ‘10’.

**Figure 6:**
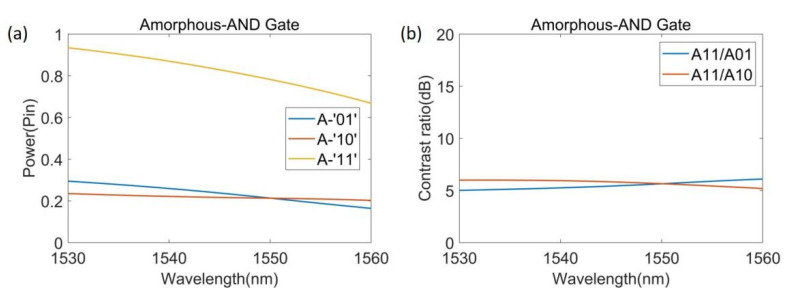
Performance graph of AND gate. (**a**) Spectral power curve of the AND gate when Sb_2_Se_3_ is in the amorphous state; (**b**) Contrast ratio curve of the AND gate.

Figure 5 shows the energy density distribution of the states of ‘10’, ‘01’, and ‘11’ when Sb_2_Se_3_ is in the amorphous state and our device functions as an AND gate. Figure 5c,d shows the energy distribution when the input state is ‘01’ and ‘10’, respectively. The output power is relatively low and the output is the ‘0’ state. When state is ‘11’, as shown in Figure 5b, the output power is high and the output is the ‘1’ state. Figure 6 is the spectral power curve of the AND gate when Sb_2_Se_3_ is in the amorphous state in the range from 1530 to 1560 nm.

Table 2 shows the output intensity of the AND gate in each source state when λ = 1550 nm. When the source state is ‘00’, the output intensity is 0 and the output state is logic ‘0’. For the source states of ‘10’, ‘01’, and ‘11’, the output intensities and states are 0.213 P_in_ and ‘1’, 0.213 P_in_ and ‘1’, and 0.783 P_in_ and ‘0’, respectively. The *CR* of the AND gate is 5.7 dB, and we set the threshold between the logics ‘0’ and ‘1’ to 0.5 P_in_. The above results show the performance parameters of our device as the XOR and the AND gates. In addition, it is worth mentioning that due to the ultra-small size of our device, its response time is less than 1 ps. These properties enable its use in photonic integrated circuits.

**Figure 7:**
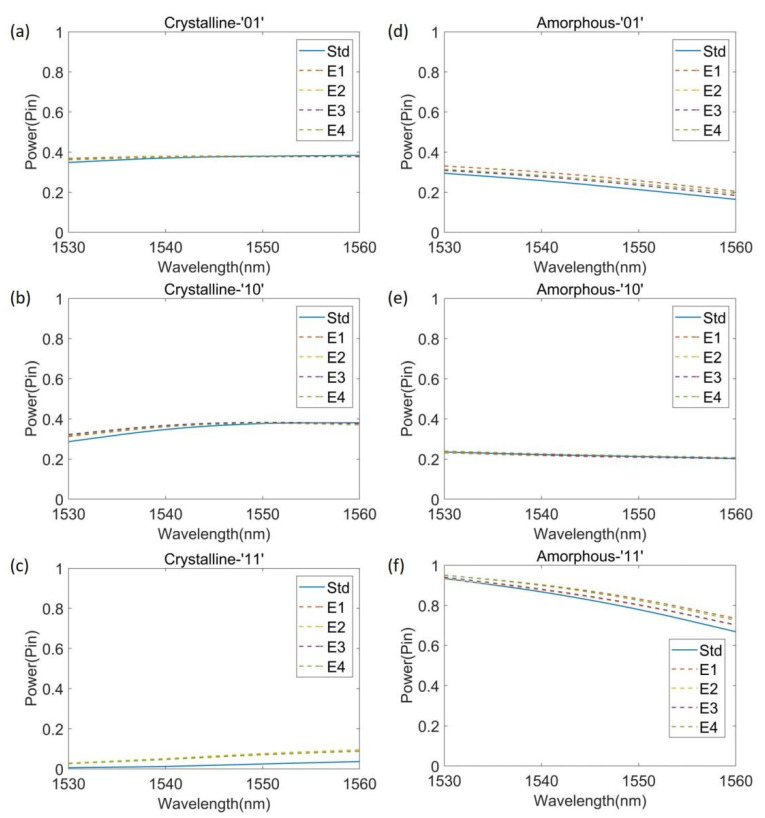
Analysis of fabrication tolerances. Comparison of the intensity between the standard device and devices with an etching error. (**a**) XOR gate with the input state of ‘01’; (**b**) XOR gate with the input state of ‘10’; (**c**) XOR gate with the input state of ‘11’; (**d**) AND gate with the input state of ‘01’; (**e**) AND gate with the input state of ‘10’; (**f**) AND gate with the input state of ‘11’.

Here, we discuss the fabrication errors and tolerance performance of our device. In actual device manufacturing, it is common that over-etching or under-etching causes errors in device manufacturing that affects device performance [58]. For our device, the etching depth should be 220 nm; however, in actual manufacturing, the etching is very likely to be insufficient, not reaching 220 nm. Therefore, we analyzed the performance of the device in the case of insufficient etching. We assumed that the depth of each etched Sb_2_Se_3_ hole was in a random range between 180–220 nm, and assumed E1, E2, E3, and E4 to be devices with errors manufactured according to this rule. Figure 7 shows the results. Figure 7a–c plot the numerical results of the XOR gate and source states of ‘01’, ‘10’, ‘11’, and the results of the AND gate are plotted in Figure 7d–f. It can be seen from Figure 7 that for the devices E1, E2, E3, and E4 with manufacturing errors, although the etching depth of each pixel is a random value between 180–220 nm, their spectrum did not change significantly, and the functionality of our device is still realized. In our standard device, the *CR* of the XOR gate is 11.8 dB, the *CR* of AND gate is 5.7 dB, as for E1, E2, E3, and E4, the lowest *CR* of the XOR gate is 6.9 dB and that of the AND gate is 5.1 dB. In this comparison, although the *CR* of the XOR gate decreased significantly, the threshold between the designed logic states of ‘0’ and ‘1’ is 0.2 P_in_, the intensity of logic ‘0’ is lower than 0.2 P_in_ and that of logic ‘1’ is higher than 0.2 P_in_. The four under-etched devices still realize the XOR gate function, as for the AND gate, the error is very small and its functioning remains virtually unchanged. The above analysis shows that our device can realize the corresponding function even if the etching depth is not up to the requirement during manufacturing, indicating that the device is robust.

**Figure 8:**
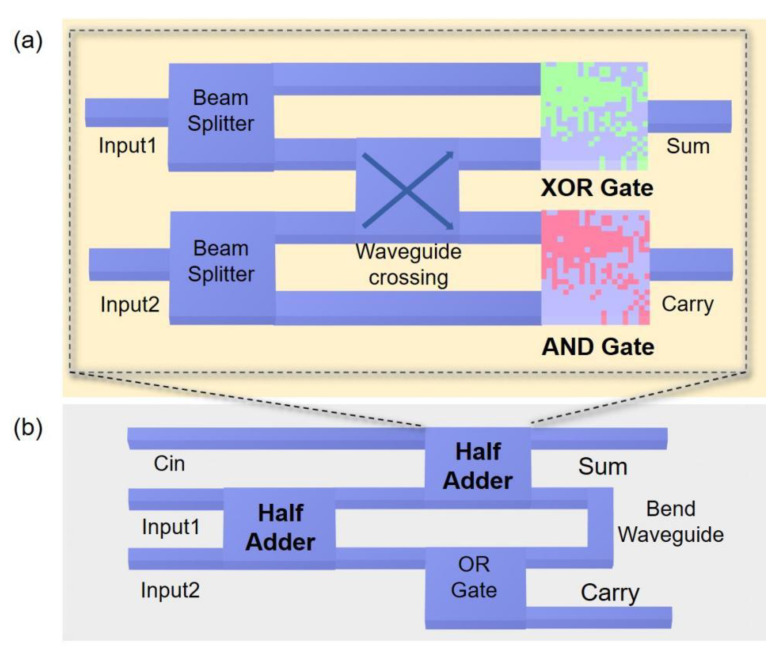
Schematic diagram of half adder and full adder composed of logic gates. (**a**) Half adder composed by two beam splitters, a waveguide crossing, a XOR gate and a AND gate; (**b**) Full adder composed by two half adder and a OR gate.

Logic gates can be combined with some common photonic devices to realize more complex devices. Here, we show two possible integration methods to realize the ultra-compact photonics half adder and full adder. As shown in Figure 8a, our logic gates, beam splitter and waveguide crossing can be combined to form a half adder. Figure 8b is a full adder, which can be composed of a half adder, OR gate and bend waveguide. Furthermore, by combining the full adders, a complete addition operation can be realized. In addition, the combinational logic circuits, encoders, and decoders similar to EICs can be implemented by logic gate devices combined with common photonic devices.

**Figure 9:**
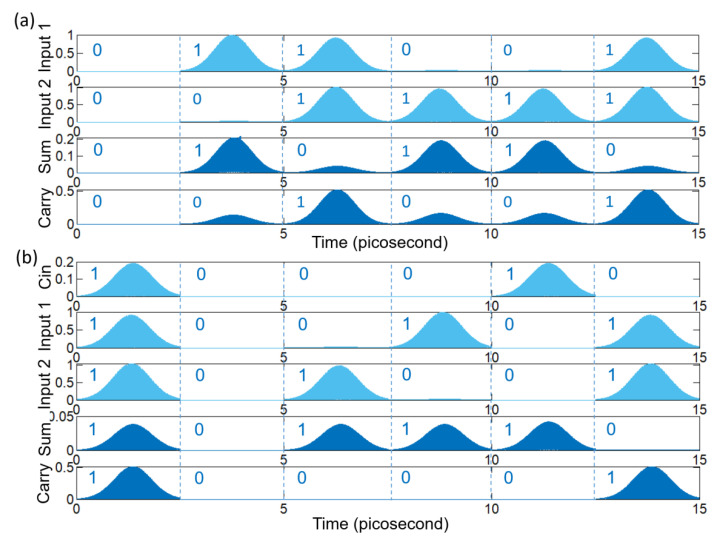
The performance of the half adder and full adder. (**a**) Input pulse and output intensity of half adder; (**b**) Input pulse and output intensity of full adder.

Furthermore, we simulated the performance of the ultra-compact photonics half adder and full adder and provide examples in Figure 9. Additionally, Figure 9a presents the example of a logic timing diagram of the half adder, where the input states are ‘00’, ‘10’, ‘11’, ‘01’, ‘01’, ‘11’, presented by 2.5 picosecond time interval pulses in sequence. The Sum output was logical ‘0’, ‘1’, ‘0’, ‘1’, ‘1’, ‘0’, respectively, and the Carry output was ‘0’, ‘0’, ‘1’, ‘0’, ‘0’, ‘1’. The full logic truth table of half adder is shown in Table 3. By comparing Table 3 and Figure 9a, we found that the photonics half adder can implement the logic functions, with a contrast ratio of the ‘1’ and ‘0’ of the Sum channel reaching 6.9 dB, and the Carry channel being 5 dB. Next, Figure 9b shows the results of full adder logic gates. In order to keep the input intensity of the half adder consistent, we set the input intensity of Cin equal to the logical ’1’ Sum output intensity of the half adder. The state of Cin, Input1 and Input2 are represented by the first bit, the second bit and the third bit, respectively. The input pulse sequence is ‘111’, ‘000’, ‘001’, ‘010’, ‘100’, ‘011’, 2.5 picosecond time per state, while the Sum output was logical ‘1’, ‘0’, ‘1’, ‘1’, ‘1’, ‘0’, respectively, and the Carry output was ‘1’, ‘0’, ‘0’, ‘0’, ‘0’, ‘1’, which is identical to the truth in in Table 4. As a result, the contrast ratio of the ‘1’ and ‘0’ of the Sum channel is about 6.9 dB, and the Carry channel is 5 dB, being similar with a half adder. Additionally, during our FDTD calculation, the example pulse interval is 2.5 picoseconds. That is, the corresponding data sequence in the photonics logic gate is 400 Giga bits per second. Moreover, if considering the extremely small size of the device, our device may support femtosecond temporal pulses, or dozens of Tera bits per second for ultra-fast data sequence.

## 4. Conclusions

In this work, we used the DBS algorithm, combined with O-PCM Sb_2_Se_3_ to design a tunable, ultra-compact silicon photonic logic gate. At 1550 nm, when Sb_2_Se_3_ is in the crystalline state, our device functions as an XOR gate, and its *CR* is 11.8 dB. When Sb_2_Se_3_ is in the amorphous state, it can work as an AND gate and has a *CR* of 5.7 dB. The footprint of our device is only 2 μm × 2 μm. We also studied the manufacturing tolerances for device etch depths ranging from 180–220 nm. With thresholds of the ‘1’ and ‘0’ states of our XOR and AND gates of 0.5 P_in_ and 0.2 P_in_, respectively, the device can still achieve corresponding functions. Furthermore, we provide a possible half adder and full adder structure, which can serve as a kind of optoelectronic fusion module. The inverse design method combined with O-PCM has great potential in the design of ultra-compact and tunable devices. We believe that such logic gates could have various application prospects in future ultra-high density, reconfigurable, scalable photonic integrated circuits.

## Figures and Tables

**Table 1:** Truth table for the all-optical XOR logic gate.

Sb_2_Se_3_ is in the Crystalline State, the Functions as an XOR Gate					
Input 1	Input 2	Output	Threshold	Output State	XOR Gate Output
0	0	0	0.2 P_in_	0	0
P_in_	0	0.381 P_in_	0.2 P_in_	1	1
0	P_in_	0.381 P_in_	0.2 P_in_	1	1
P_in_	P_in_	0.025 P_in_	0.2 P_in_	0	0

**Table 2:** Truth table for the all-optical AND logic gate.

Sb_2_Se_3_ is in the Amorphous State and Functions as an AND Gate					
Input1	Input2	Output	Threshold	Output Logic State	AND Gate Output
0	0	0	0.5 P_in_	0	0
P_in_	0	0.213 P_in_	0.5 P_in_	0	0
0	P_in_	0.213 P_in_	0.5 P_in_	0	0
P_in_	P_in_	0.783 P_in_	0.5 P_in_	1	1

**Table 3:** Logic truth table of the half adder.

Input1	0	1	0	1
Input2	0	0	1	1
Sum	0	1	1	0
Carry	0	0	0	1

**Table 4:** Logic truth table of the full adder.

Cin	0	0	0	1	0	1	1	1
Input1	0	0	1	0	1	0	1	1
Input2	0	1	0	0	1	1	0	1
Sum	0	1	1	1	0	0	0	1
Carry	0	0	0	0	1	1	1	1

## Data Availability

Data available in a publicly accessible repository.

## References

[1] Koos C., Vorreau P., Vallaitis T., Dumon P., Bogaerts W., Baets R., Esembeson B., Biaggio I., Michinobu T., Diederich F. (2009). All-optical high-speed signal processing with silicon–organic hybrid slot waveguides. Nat. Photonics.

[2] Miller D.A. (2010). Are optical transistors the logical next step?. Nat. Photonics.

[3] Chu K.-C., Chang K.-C., Wang H.-C., Lin Y.-C., Hsu T.-L. (2020). Field-programmable gate array-based hardware design of optical fiber transducer integrated platform. J. Nanoelectron. Optoelectron..

[4] Menezes J., De Fraga W., Ferreira A., Saboia K., Guimarães G., Sousa J., Rocha H., Sombra A. (2007). Logic gates based in two-and three-modes nonlinear optical fiber couplers. Opt. Quantum Electron..

[5] Jasim M.A., Aldalbahi A. (2019). Design of XOR photonic gate using highly nonlinear fiber. Electronics.

[6] Gayen D.K., Chattopadhyay T. (2013). Designing of optimized all-optical half adder circuit using single quantum-dot semiconductor optical amplifier assisted Mach-Zehnder interferometer. J. Lightwave Technol..

[7] Kaur S., Kaler R.-S. (2012). Ultrahigh speed reconfigurable logic operations based on single semiconductor optical amplifier. J. Opt. Soc. Korea.

[8] Ma S., Chen Z., Sun H., Dutta N.K. (2010). High speed all optical logic gates based on quantum dot semiconductor optical amplifiers. Opt. Express.

[9] Mehdizadeh F., Soroosh M. (2016). Designing of all optical NOR gate based on photonic crystal. Indian J. Pure Appl. Phys. (IJPAP).

[10] Liu Y., Qin F., Meng Z.-M., Zhou F., Mao Q.-H., Li Z.-Y. (2011). All-optical logic gates based on two-dimensional low-refractive-index nonlinear photonic crystal slabs. Opt. Express.

[11] Ghadrdan M., Mansouri-Birjandi M.A. (2013). Concurrent implementation of all-optical half-adder and AND & XOR logic gates based on nonlinear photonic crystal. Opt. Quantum Electron..

[12] Parandin F., Malmir M.R., Naseri M., Zahedi A. (2018). Reconfigurable all-optical NOT, XOR, and NOR logic gates based on two dimensional photonic crystals. Superlattices Microstruct..

[13] Caballero L.E.P., Cano J.P.V., Guimaraes P.S., Neto O.P.V. (2017). Effect of structural disorder on photonic crystal logic gates. IEEE Photonics J..

[14] Zaghloul Y., Zaghloul A. (2006). Complete all-optical processing polarization-based binary logic gates and optical processors. Opt. Express.

[15] Mohebzadeh-Bahabady A., Olyaee S. (2018). All-optical NOT and XOR logic gates using photonic crystal nano-resonator and based on an interference effect. IET Optoelectron..

[16] Jiang Y.-C., Liu S.-B., Zhang H.-F., Kong X.-K. (2014). Reconfigurable design of logic gates based on a two-dimensional photonic crystals waveguide structure. Opt. Commun..

[17] Molesky S., Lin Z., Piggott A.Y., Jin W., Vucković J., Rodriguez A.W. (2018). Inverse design in nanophotonics. Nat. Photonics.

[18] Huang J., Ma H., Chen D., Yuan H., Zhang J., Li Z., Han J., Wu J., Yang J. (2021). Digital nanophotonics: The highway to the integration of subwavelength-scale photonics. Nanophotonics.

[19] Ma H., Huang J., Zhang K., Yang J. (2020). Arbitrary-direction, multichannel and ultra-compact power splitters by inverse design method. Opt. Commun..

[20] Ma H., Huang J., Zhang K., Yang J. (2020). Inverse-designed arbitrary-input and ultra-compact 1× N power splitters based on high symmetric structure. Sci. Rep..

[21] Huang J., Yang J., Chen D., Bai W., Han J., Zhang Z., Zhang J., He X., Han Y., Liang L. (2020). Implementation of on-chip multi-channel focusing wavelength demultiplexer with regularized digital metamaterials. Nanophotonics.

[22] Yuan H., Huang J., Wang Z., Zhang J., Deng Y., Lin G., Wu J., Yang J. (2021). An ultra-compact dual-channel multimode wavelength demultiplexer based on inverse design. Results Phys..

[23] Sapra N.V., Vercruysse D., Su L., Yang K.Y., Skarda J., Piggott A.Y., Vučković J. (2019). Inverse design and demonstration of broadband grating couplers. IEEE J. Sel. Top. Quantum Electron..

[24] Piggott A.Y., Lu J., Babinec T.M., Lagoudakis K.G., Petykiewicz J., Vučković J. (2014). Inverse design and implementation of a wavelength demultiplexing grating coupler. Sci. Rep..

[25] Shen B., Wang P., Polson R., Menon R. (2015). An integrated-nanophotonics polarization beamsplitter with 2.4 × 2.4 μm^2^ footprint. Nat. Photonics.

[26] Yu Z., Feng A., Xi X., Sun X. (2019). Inverse-designed low-loss and wideband polarization-insensitive silicon waveguide crossing. Opt. Lett..

[27] Liu Y., Xu K., Wang S., Shen W., Xie H., Wang Y., Xiao S., Yao Y., Du J., He Z. (2019). Arbitrarily routed mode-division multiplexed photonic circuits for dense integration. Nat. Commun..

[28] Kim G., Menon R. (2014). An ultra-small three dimensional computational microscope. Appl. Phys. Lett..

[29] Sanchis P., Villalba P., Cuesta F., Håkansson A., Griol A., Galán J.V., Brimont A., Martí J. (2009). Highly efficient crossing structure for silicon-on-insulator waveguides. Opt. Lett..

[30] Yu Z., Cui H., Sun X. (2017). Genetically optimized on-chip wideband ultracompact reflectors and Fabry–Perot cavities. Photonics Res..

[31] Mak J.C., Sideris C., Jeong J., Hajimiri A., Poon J.K. (2016). Binary particle swarm optimized 2 × 2 power splitters in a standard foundry silicon photonic platform. Opt. Lett..

[32] Lu Q., Yan X., Wei W., Zhang X., Zhang M., Zheng J., Li B., Lin Q., Ren X. (2019). High-speed ultra-compact all-optical NOT and AND logic gates designed by a multi-objective particle swarm optimized method. Opt. Laser Technol..

[33] Lu J., Vučković J. (2010). Inverse design of nanophotonic structures using complementary convex optimization. Opt. Express.

[34] Su L., Piggott A.Y., Sapra N.V., Petykiewicz J., Vuckovic J. (2018). Inverse design and demonstration of a compact on-chip narrowband three-channel wavelength demultiplexer. ACS Photonics.

[35] Hammond A.M., Camacho R.M. (2019). Designing integrated photonic devices using artificial neural networks. Opt. Express.

[36] So S., Badloe T., Noh J., Bravo-Abad J., Rho J. (2020). Deep learning enabled inverse design in nanophotonics. Nanophotonics.

[37] Tahersima M.H., Kojima K., Koike-Akino T., Jha D., Wang B., Lin C., Parsons K. (2019). Deep neural network inverse design of integrated photonic power splitters. Sci. Rep..

[38] Miller K.J., Haglund R.F., Weiss S.M. (2018). Optical phase change materials in integrated silicon photonic devices. Opt. Mater. Express.

[39] Abdollahramezani S., Hemmatyar O., Taghinejad H., Krasnok A., Kiarashinejad Y., Zandehshahvar M., Alù A., Adibi A. (2020). Tunable nanophotonics enabled by chalcogenide phase-change materials. Nanophotonics.

[40] Gerislioglu B., Bakan G., Ahuja R., Adam J., Mishra Y.K., Ahmadivand A. (2020). The role of Ge_2_Sb_2_Te_5_ in enhancing the performance of functional plasmonic devices. Mater. Today Phys..

[41] Miller K.J., Hallman K.A., Haglund R.F., Weiss S.M. (2017). Silicon waveguide optical switch with embedded phase change material. Opt. Express.

[42] Zhang H., Zhou L., Lu L., Xu J., Wang N., Hu H., Rahman B.A., Zhou Z., Chen J. (2019). Miniature multilevel optical memristive switch using phase change material. ACS Photonics.

[43] Zhang C., Zhang M., Xie Y., Shi Y., Kumar R., Panepucci R.R., Dai D. (2020). Wavelength-selective 2× 2 optical switch based on a Ge_2_Sb_2_Te_5_-assisted microring. Photonics Res..

[44] Yang X., Nisar M.S., Yuan W., Zheng F., Lu L., Chen J., Zhou L. (2021). Phase change material enabled 2 × 2 silicon nonvolatile optical switch. Opt. Lett..

[45] Bai W., Yang P., Huang J., Chen D., Zhang J., Zhang Z., Yang J., Xu B. (2019). Near-infrared tunable metalens based on phase change material Ge_2_Sb_2_Te_5_. Sci. Rep..

[46] Tseng M.L., Hsiao H.H., Chu C.H., Chen M.K., Sun G., Liu A.Q., Tsai D.P. (2018). Metalenses: Advances and applications. Adv. Opt. Mater..

[47] Qin S., Xu N., Huang H., Jie K., Liu H., Guo J., Meng H., Wang F., Yang X., Wei Z. (2021). Near-infrared thermally modulated varifocal metalens based on the phase change material Sb_2_S_3_. Opt. Express.

[48] Chen H., Jia H., Wang T., Tian Y., Yang J. (2020). Broadband Nonvolatile Tunable Mode-Order Converter Based on Silicon and Optical Phase Change Materials Hybrid Meta-Structure. J. Lightwave Technol..

[49] Mishra A.K., Lahiri B., Philip J. (2019). Superior thermal conductivity and photo-thermal conversion efficiency of carbon black loaded organic phase change material. J. Mol. Liq..

[50] Wu C., Yu H., Lee S., Peng R., Takeuchi I., Li M. (2021). Programmable phase-change metasurfaces on waveguides for multimode photonic convolutional neural network. Nat. Commun..

[51] Zhou Y., Zheng S., Zhang G. (2019). Artificial neural network based multivariable optimization of a hybrid system integrated with phase change materials, active cooling and hybrid ventilations. Energy Convers. Manag..

[52] Bakan G., Gerislioglu B., Dirisaglik F., Jurado Z., Sullivan L., Dana A., Lam C., Gokirmak A., Silva H. (2016). Extracting the temperature distribution on a phase-change memory cell during crystallization. J. Appl. Phys..

[53] Kato K., Kuwahara M., Kawashima H., Tsuruoka T., Tsuda H. (2017). Current-driven phase-change optical gate switch using indium–tin-oxide heater. Appl. Phys. Express.

[54] Qihang Z., Yifei Z., Junying L., Richard S., Tian G., Juejun H. (2018). Broadband nonvolatile photonic switching based on optical phase change materials: Beyond the classical figure-of-merit. Opt. Lett..

[55] Delaney M., Zeimpekis I., Du H., Yan X., Banakar M., Thomson D.J., Hewak D.W., Muskens O.L. (2021). Nonvolatile programmable silicon photonics using an ultralow-loss Sb_2_Se_3_ phase change material. Sci. Adv..

[56] Delaney M., Zeimpekis I., Lawson D., Hewak D.W., Muskens O.L. (2020). A new family of ultralow loss reversible phase-change materials for photonic integrated circuits: Sb_2_S_3_ and Sb_2_Se_3_. Adv. Funct. Mater..

[57] Chen C., Bobela D.C., Yang Y., Lu S., Zeng K., Ge C., Yang B., Gao L., Zhao Y., Beard M.C. (2017). Characterization of basic physical properties of Sb_2_Se_3_ and its relevance for photovoltaics. Front. Optoelectron..

[58] Lu L., Liu D., Zhou F., Li D., Cheng M., Deng L., Fu S., Xia J., Zhang M. (2016). Inverse-designed single-step-etched colorless 3 dB couplers based on RIE-lag-insensitive PhC-like subwavelength structures. Opt. Lett..

